# A new cryptic species of *Nagiella* Munroe from China revealed by DNA barcodes and morphological evidence (Lepidoptera, Crambidae, Spilomelinae)

**DOI:** 10.3897/zookeys.679.11960

**Published:** 2017-06-08

**Authors:** Misbah Ullah, Zhaofu Yang, Pingping Qiao, Yalin Zhang

**Affiliations:** 1 Key laboratory of Plant Protection Resources and Pest Management, Ministry of Education; Entomological Museum, College of Plant Protection, Northwest A&F University, Yangling, Shaanxi 712100, China

**Keywords:** COI gene, genitalia, *Scopula quadrimaculalis*, taxonomy

## Abstract

*Nagiella occultalis* Misbah & Yang, **sp. n**. from China is described and illustrated. This new species is very similar to *N. quadrimaculalis* (Kollar, 1844) in general morphological characters of forewing and male genitalia. Molecular evidence shows that these two species diverge in COI barcode region by more than 3.2%. Sequence divergence among the two species is congruent with subtle morphological differences. Wing venation and male genitalia of the two species are compared and illustrated.

## Introduction

The subfamily Spilomelinae (Crambidae) is the largest subfamily of pyraloid moths including about 3300 species in more than 300 genera having worldwide distribution (Munroe and Solis 1999). The genus *Nagiella* Munroe, 1976 is one of the less speciose genera of Spilomelinae (Munroe 1976). Compared to other genera of this subfamily and despite its small size, *Nagiella* has been little studied and no comprehensive studies have been made on the taxonomy of its constituent species. The only taxonomic efforts were made by Munroe in 1976. This genus was originally described as *Nagia* by Walker in 1866 based on the type species *Nagia desmialis* Walker, 1866. Munroe (1976) recognized that *Nagia* Walker, 1866 is a junior homonym of *Nagia* Walker, 1858 (Lepidoptera: Noctuidae) and replaced it with the new name *Nagiella* Munroe, 1976. This genus is widely distributed in Malaysia (Borneo and Sarawak), Burma, China, and Japan (Munroe 1976; Inoue 1982; Wang 1980). The genus comprises three described species: *Nagiella inferior* (Hampson, 1898), *Nagiella quadrimacualis* (Kollar, 1844) with two junior subjective synonyms, *desmialis* Walker, 1866 and *incomitata* Swinhoe, 1894, and *Nagiella hortulatoides* Munroe, 1976 distributed in northeastern Burma. The generic characters as defined by Munroe (1976) are: uncus truncate, short and wide; gnathos ribbon-like; subscaphium elongate; valva broader with stout setae subapically, sella digitiform, elongate and sharp; cornutus absent. This provides the baseline description of the genus on which the present study is based.

Recently the integration of DNA barcoding and morphological approaches opened the field for researchers in accelerating species identification and assisted in detecting previously undetected cryptic species (Sutrisno 2005; Mutanen et al. 2012; Haines & Rubinoff 2012; Yang et al. 2012; Rajaeish et al. 2013; Yang et al. 2016; Mally et al. 2016). The taxonomic placement of *N. occultalis* sp. n. has been unclear; therefore, an integrative approach was designed to study the generic differences (Munroe 1976). In the present integrative taxonomic study, *N. occultalis* sp. n. collected from Shaanxi and Hubei Province, China, is described.

## Materials and methods

### Taxon sampling

Three specimens of *N. occultalis* sp. n. were collected from Taibai Mountain, Shaanxi and Wufeng, Hubei in China and 15 specimens of *N. quadrimaculalis* were collected from various localities (Table 1). Genitalia preparation mainly follows Landry (2007) and Yang et al. (2012) and terminology follows Kristensen (2003). The images of adults and genitalia were captured with a Canon Power Shot SX60 digital camera and (ZEISS Discovery V20) stereomicroscope equipped with an AxioCam ICc5 camera, respectively and measurement was taken in mm by scale bar equipped in stereomicroscope. Type material of the new species is deposited in the Entomological Museum, College of Plant Protection, Northwest A&F University, Yangling, Shaanxi, China (NWAFU).

**Table 1.** Specimens of two *Nagiella* species from China examined in this study.

Identification	BIN	Process ID	Sample ID	Length of sequence (bp)	GenBank Accession	Province	Genitalia slide number
*N. occultalis* sp. n.	BOLD:AAD8179	CNPYB439-16	NAFU Pyr002290	658	KY080696	Shaanxi	
*N. occultalis* sp. n.	BOLD:AAD8179	CNPYB407-16	NAFU Pyr002397	658	KY080703	Shaanxi	NAFU Pyr002065
*N. occultalis* sp. n.	BOLD:AAD8179	CNPYD499-10	Pyr000499	658	HM908668	Hubei	
*N. quadrimaculalis*		CNPYA401-10	NAFU Pyr000401	0		Yunnan	
*N. quadrimaculalis*		CNPYA402-10	NAFU Pyr000402	0		Sichuan	
*N. quadrimaculalis*		CNPYA403-10	NAFU Pyr000403	0		Yunnan	
*N. quadrimaculalis*		CNPYA404-10	NAFU Pyr000404	0		Yunnan	
*N. quadrimaculalis*		CNPYB409-16	NAFU Pyr002070	0		Shaanxi	NAFU Pyr002070
*N. quadrimaculalis*		CNPYB410-16	NAFU Pyr002261	0		Shaanxi	NAFU Pyr002261
*N. quadrimaculalis*		CNPYB411-16	NAFU Pyr002262	0		Shaanxi	
*N. quadrimaculalis*	BOLD:AAD8178	CNPYB412-16	NAFU Pyr002263	658	KY080700	Shaanxi	
*N. quadrimaculalis*	BOLD:AAD8178	CNPYB413-16	NAFU Pyr002264	658	KY080702	Shaanxi	
*N. quadrimaculalis*	BOLD:AAD8178	CNPYB414-16	NAFU Pyr002265	658	KY080704	Shaanxi	
*N. quadrimaculalis*	BOLD:AAD8178	CNPYB415-16	NAFU Pyr002266	658	KY080698	Shaanxi	
*N. quadrimaculalis*	BOLD:AAD8178	CNPYB416-16	NAFU Pyr002267	658	KY080694	Shaanxi	
*N. quadrimaculalis*	BOLD:AAD8178	CNPYB417-16	NAFU Pyr002268	658	KY080705	Shaanxi	
*N. quadrimaculalis*	BOLD:AAD8178	CNPYB418-16	NAFU Pyr002269	658	KY080697	Shaanxi	
*N. quadrimaculalis*		CNPYB419-16	NAFU Pyr002270	0		Shaanxi	
*N. quadrimaculalis*		CNPYB420-16	NAFU Pyr002271	0		Shaanxi	
*N. quadrimaculalis*		CNPYB421-16	NAFU Pyr002272	0		Henan	NAFU Pyr002272
*N. quadrimaculalis*		CNPYB422-16	NAFU Pyr002273	0		Henan	NAFU Pyr002273
*N. quadrimaculalis*		CNPYB423-16	NAFU Pyr002274	0			
*N. quadrimaculalis*		CNPYB424-16	NAFU Pyr002275	0		Hunan	
*N. quadrimaculalis*		CNPYB425-16	NAFU Pyr002276	0		Hunan	
*N. quadrimaculalis*		CNPYB426-16	NAFU Pyr002277	0			
*N. quadrimaculalis*		CNPYB427-16	NAFU Pyr002278	0		Fujian	
*N. quadrimaculalis*		CNPYB428-16	NAFU Pyr002279	0		Hainan	
*N. quadrimaculalis*		CNPYB429-16	NAFU Pyr002280	0		Hainan	
*N. quadrimaculalis*		CNPYB430-16	NAFU Pyr002281	0			
*N. quadrimaculalis*		CNPYB431-16	NAFU Pyr002282	0		Zhejiang	
*N. quadrimaculalis*		CNPYB432-16	NAFU Pyr002283	0		Yunnan	
*N. quadrimaculalis*		CNPYB433-16	NAFU Pyr002284	0			NAFU Pyr002284
*N. quadrimaculalis*		CNPYB434-16	NAFU Pyr002285	0			
*N. quadrimaculalis*		CNPYB435-16	NAFU Pyr002286	0			
*N. quadrimaculalis*		CNPYB436-16	NAFU Pyr002287	0			NAFU Pyr002287
*N. quadrimaculalis*		CNPYB437-16	NAFU Pyr002288	0			
*N. quadrimaculalis*	BOLD:AAD8178	CNPYB438-16	NAFU Pyr002289	658	KY080695	Shaanxi	
*N. quadrimaculalis*	BOLD:AAD8178	CNPYB440-16	NAFU Pyr002291	658	KY080701	Shaanxi	NAFU Pyr002291
*N. quadrimaculalis*	BOLD:AAD8178	CNPYB441-16	NAFU Pyr002292	658	KY080699	Shaanxi	
*N. quadrimaculalis*		CNPYB408-16	NAFU Pyr002398	0		Shaanxi	NAFU Pyr002067
*N. quadrimaculalis*	BOLD:AAD8178	CNPYD497-10	Pyr000497	622	HM908666	Hubei	
*N. quadrimaculalis*	BOLD:AAD8178	CNPYD498-10	Pyr000498	658	HM908667	Hubei	
*N. quadrimaculalis*		CNPYD500-10	Pyr000500	0		Hubei	
*N. quadrimaculalis*		CNPYD501-10	Pyr000501	0		Hubei	
*N. quadrimaculalis*		CNPYD502-10	Pyr000502	0		Hubei	
*N. quadrimaculalis*	BOLD:AAD8178	CNPYD503-10	Pyr000503	658	HM908669	Hubei	
*N. quadrimaculalis*	BOLD:AAD8178	CNPYD504-10	Pyr000504	658	HM908670	Sichuan	
*N. quadrimaculalis*	BOLD:AAD8178	CNPYD505-10	Pyr000505	658	HM908671	Sichuan	

**Table 2.** Kimura 2-parameter genetic distances calculated within (in italic) and between three species of *Nagiella*.

	*Nagiella occultalis* sp. n.	*Nagiella quadrimaculalis*	*Nagiella inferior*	*Patania ruralis* (outgroup)
*Nagiella occultalis* sp. n.	**0.0000000**	0.0072358	0.0086344	
*Nagiella quadrimaculalis*	0.0320975	**0.000787822**	0.0101216	
*Nagiella inferior*	0.0475427	0.0598071	**0.000761036**	
*Patania ruralis* (Outgroup)	0.1156349	0.1165689	0.1134248	**0.009202714**

The diagonal row of values (in bold) indicates intra specific distances, the values below the diagonal indicates mean interspecific distances and values above the diagonal indicates SE estimates obtained by bootstrap procedure (1000 replicates) as implemented in MEGA 6.0. The three species were defined using the 2.0% divergence.

### DNA extraction, PCR amplification, and sequencing

Genomic DNA was extracted from insect legs by following the method of Ivanova et al. (2006). PCR amplifications were conducted to amplify a full-length (658 bp) barcode region of the mitochondrial COI gene by the primers pairs, *LepF1* and *LepR1* (Hajibabaei et al. (2006). After the PCR products were checked with 1% agarose gel, sequencing was performed at Sangon Biotechnology Co., Ltd. (Shanghai, China) using the same primers as in PCR.

### Data analysis

Sequence alignment was carried out by using MUSCLE algorithm implemented in MEGA 6.0 (Tamura et al. 2013). MEGA 6.0 was also used to perform genetic distances under the Kimura 2-parameter model of base substitution, to produce the Neighbor-Joining (NJ) tree, and to perform bootstrap analysis (1000 replicates) (Kimura 1980). In the present study, we included four sequences of *Nagiella inferior* and selected *Patania ruralis* (Scopoli, 1763) as the primary out-group to build the tree which is most closely related genus. Sequences obtained from the current study were deposited in GenBank, in addition to being available in the BOLD dataset DS-PLEQUA.

## Results

### DNA sequence analysis

**Figure 1.**
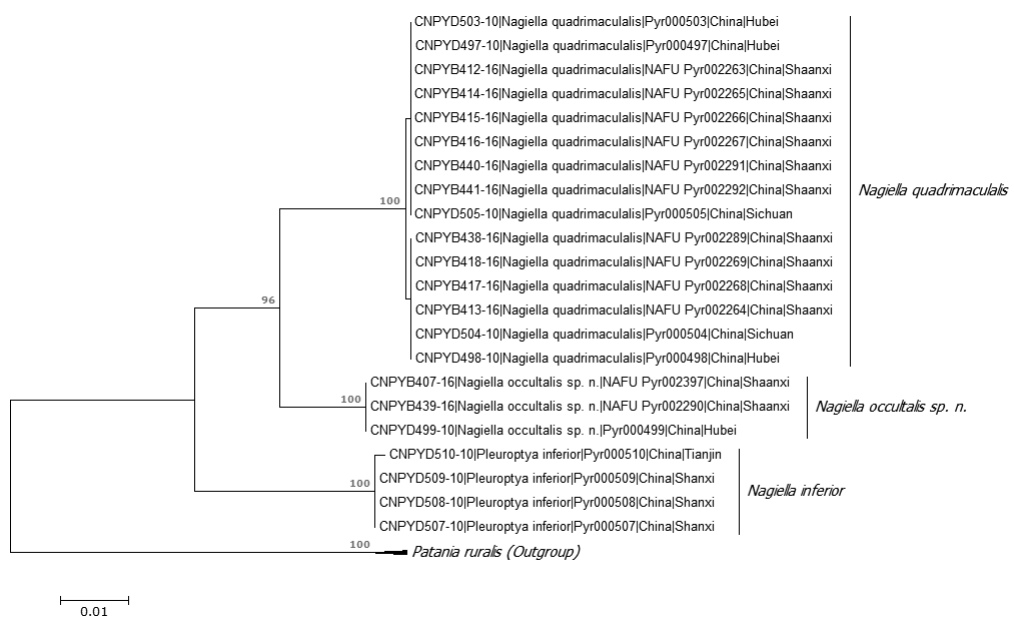
Neighbor-joining tree (K2P) based on the 22 COI sequences of the three *Nagiella* species from China, rooted with *Patania ruralis* as outgroup. Bootstrap values <75 are not shown.

A total of 18 COI gene sequences of *N. occultalis* sp. n. and *N. quadrimaculalis* were obtained. The lengths were from 622–658 bp (mean 656 bp). The genetic distances within and between these two species of *Nagiella* are given in Table 2. Intraspecific genetic divergences ranged from 0.00–0.16 % (mean 0.078 %), whereas interspecific genetic divergence ranged from 3.12–3.28 % (mean 3.21 %). The neighbor-joining (NJ) tree (Fig. 1) showed two distinct barcode clusters that correspond to morphological differences between these two species.

### Taxonomy

#### Nagiella occultalis

Taxon classificationAnimaliaLepidopteraCrambidae

Misbah & Yang sp. n.

http://zoobank.org/C252DFC4-FA47-4A75-85CE-3D7E99E25177

##### Etymology.

The specific epithet refers to “cryptic”, as this previously undetected species stood within the *N. quadrimaculalis* complex.

##### Diagnosis.

This species can be distinguished from *N. quadrimaculalis* by the width and length of the uncus, the proportions of the valva and transtilla, and size of the forewing, as described in Table 3.

##### Description

**Figure 2.**
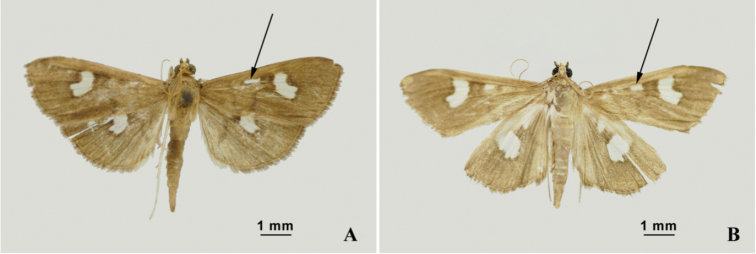
Adults, dorsal aspect **A** *N. occultalis* sp. n. **B** *N. quadrimaculalis*.

**Figure 3.**
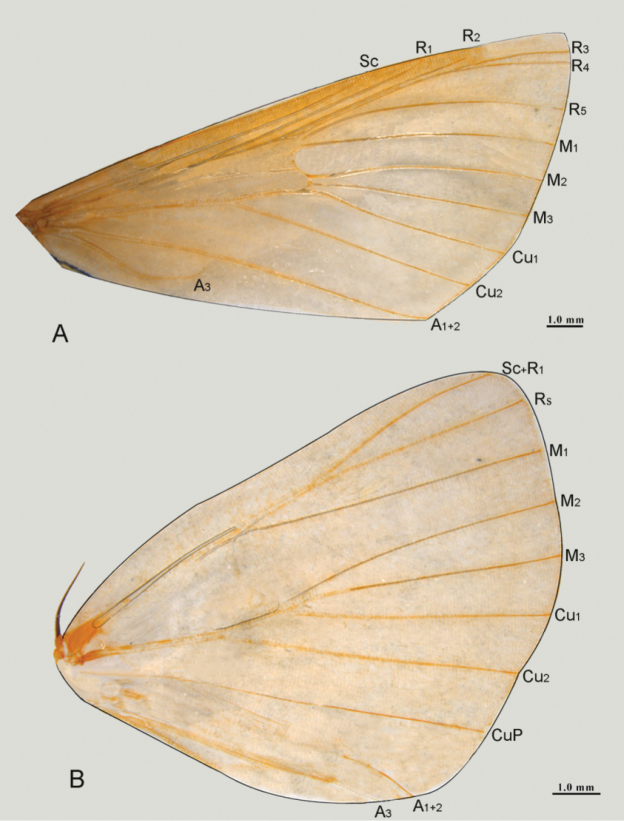
Wing venation of *N. occultalis* sp. n.

**(Figs 2A, 3).** Body yellowish brown to black with white patches on wings. Length of forewing 15–16 mm. Head with frons shiny white, labial palpus bent over top of head. Patagium shiny black. Forewing dark brown, with small bean-shaped white spot of varying size near middle of reniform stigma in the base of discal cell; rectangular subdiscal white spot proportionally narrower or elongate. R_1_ arising from cell at about apical third and almost parallel to Sc, R_2_ parallel to R_1_ but close to R_3+4_. R_3_ and R_4_ long stalked and reached apical margin. M_2_ and M_3_ closer to each other at base than M_1_ (almost of the same length) but all median veins on equal distance on outer margin. Vein Cu_2_ originating from 2/3 of the cell. Anal vein A_1+2_ prominent and complete while A_3_ diminished before mid-length of wing. Hind wing with bean-shaped white spots near outer margin of medial line at terminal part of discal cell; Sc, radial and M_1_ on same stalk, anal vein A_3_ incomplete.

**Figure 4.**
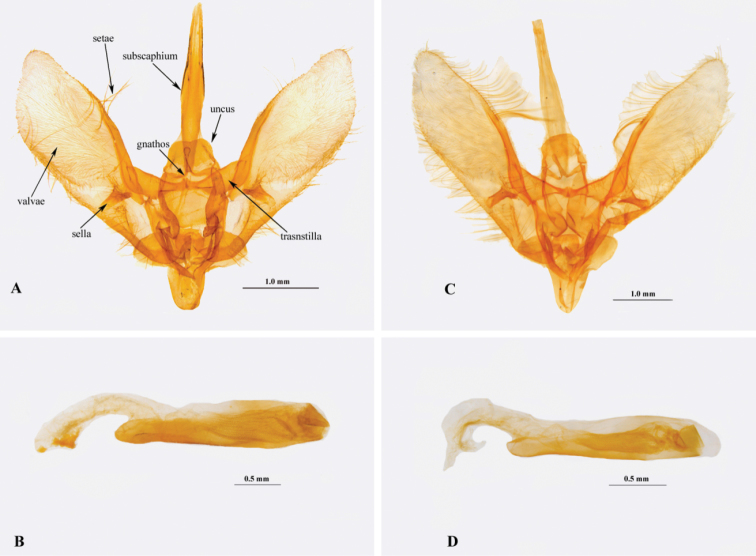
Male genitalia **A, B** *N. occultalis* sp. n., genitalia slide NAFU PYR 002397 **C, D** *N. quadrimaculalis*, genitalia slide NAFU PYR 002069.

**Male genitalia (Fig. 4A, B).** Uncus subtrapezoid in outline, posterolateral angles rounded, distal margin slightly notched medially. Gnathos with proximal arms extended transversely from teguminal margin and joined mesially into subclavate distal projection extended almost to level of apex of uncus. Subscaphium very elongate, apex extended beyond apex of valvae. Transtilla triangular, broad basally and apically narrower. Valva relatively short and broad with several thickened setae on posterior margin. Sella elongate, digitiform, straight laterally, apex rounded. Saccus roundly conical. Phallus cylindrical, terminal end somewhat tapered, cornutus absent.

**Female.** Unknown

##### Distribution.

China (Taibai Mountain, Shaanxi; Wufeng, Hubei).

##### Type material.

**Holotype.** ♂: China: Shaanxi, Taibai Mountain, 1051 m, 25 July 2014, Zhou Lin (NWAFU), Specimen ID: NAFU PYR002397. Genitalia slide number: NAFU PYR002397. **Paratypes.** 1 ♂, same data as the holotype except 24 July 2014; 1 ♂, China, Hubei, Wufeng, Changleping town, 14 July 2008, Zhao Lu.

##### Remarks.

The genus *Nagiella*, formerly comprised of three recognized species widespread in Burma, China, Japan and Malaysia (Borneo and Sarawak), is now increased to four with *N. occultalis* sp. n.

**Table 3.** Morphological differences between *Nagiella occultalis* sp. n. and *N. qudrimaculalis*.

Characteristics	*N. occultalis* sp. n.	*N. quadrimaculalis*
Forewing length	15–16 mm (Fig. 2A)	18–20 mm (Fig. 2B)
Small subdiscal spot on forewing	Proportionally narrower or elongate	Sub-quadrate
Uncus width and length	0.4 × 0.6 mm (Fig. 4A)	0.3 × 0.68 mm (Fig. 4C)
Posterior margin of uncus	Slightly notched medially	Evenly rounded
Valva	Broader, W/L 0.91 × 3.09 mm	Slender, W/L 0.7 mm × 2.08 mm
Sella with ventral edge	Straight	Slightly incurved
Subscaphium	Elongate, conical sclerotized	Unsclerotized
Size of transtilla	Narrower, 0.28 × 0.8 mm	Broadly triangular 0.3 × 0.9 mm
Phallus	Phallus L/valva L ratio 1.19 (Fig. 4B)	Phallus L/valva L ratio 1.7 (Fig. 4D)

## Discussion

Munroe (1976) indicated that *Nagiella* differs from *Pleuroptya* Meyrick, 1890 in several genital characters, i.e. short, wide uncus, gnathos developed, cornutus absent, valva broader with stout setae subapically, as well as in type of wing maculation. This taxonomic treatment was followed by Kirti and Sodhi (2001) and Rose (2002). However, members of the genus *Nagiella* have been placed in various genera, namely *Pleuroptya* Meyrick, 1890, *Syllepte* Hübner, 1823, *Patania* Moore, 1888 (Inoue 1982; Wang, 1980; Li et al. 2009; Xu 2015; Irungbam et al. 2016; Kirti et al. 2016). Leraut (1997) also listed *Nagiella* as a junior synonym of *Pleuroptya*. Kirti and Gill (2007) synonymized *Pleuroptya* Meyrick, 1890 under *Patania* on the basis of shared characters such as the lack of gnathos, the valvae leaf-like and without setae, and the presence of distinct cornuti present in the phallus. In *Nagiella* the gnathos is present, the valvae are broader and bear stout subapical setae, and the cornuti are absent. Based on this morphological evidence and online Lepindex (Beccaloni et al. 2003), we consider that *Nagiella* warrants distinct generic status and we re-instate it as valid.

## Supplementary Material

XML Treatment for Nagiella occultalis
